# Environmental and anthropogenic drivers of connectivity patterns: A basis for prioritizing conservation efforts for threatened populations

**DOI:** 10.1111/eva.12443

**Published:** 2016-12-20

**Authors:** Chrysoula Gubili, Stefano Mariani, Byron V. Weckworth, Paul Galpern, Allan D. McDevitt, Mark Hebblewhite, Barry Nickel, Marco Musiani

**Affiliations:** ^1^ School of Environment and Life SciencesUniversity of SalfordSalfordUK; ^2^ Faculties of Environmental Design and Veterinary MedicineUniversity of CalgaryCalgaryABCanada; ^3^ PantheraNew YorkNYUSA; ^4^ Wildlife Biology ProgramDepartment of Ecosystem and Conservation SciencesCollege of Forestry and ConservationUniversity of MontanaMissoulaMTUSA; ^5^ Environmental Studies DepartmentCenter for Integrated Spatial ResearchUniversity of CaliforniaSanta CruzCAUSA

**Keywords:** connectivity, gene flow, isolation, landscape genetics, nuclear loci, *Rangifer tarandus*, reciprocal causal modelling

## Abstract

Ecosystem fragmentation and habitat loss have been the focus of landscape management due to restrictions on contemporary connectivity and dispersal of populations. Here, we used an individual approach to determine the drivers of genetic differentiation in caribou of the Canadian Rockies. We modelled the effects of isolation by distance, landscape resistance and predation risk and evaluated the consequences of individual migratory behaviour (seasonally migratory vs. sedentary) on gene flow in this threatened species. We applied distance‐based and reciprocal causal modelling approaches, testing alternative hypotheses on the effects of geographic, topographic, environmental and local population‐specific variables on genetic differentiation and relatedness among individuals. Overall, gene flow was restricted to neighbouring local populations, with spatial coordinates, local population size, groups and elevation explaining connectivity among individuals. Landscape resistance, geographic distances and predation risk were correlated with genetic distances, with correlations threefold higher for sedentary than for migratory caribou. As local caribou populations are increasingly isolated, our results indicate the need to address genetic connectivity, especially for populations with individuals displaying different migratory behaviours, whilst maintaining quality habitat both within and across the ranges of threatened populations.

## 1 Introduction

The current global biodiversity crisis (Pimm, Russell, Gittleman, & Brooks, 1995) is partially attributed to habitat loss and degradation (Turner et al., 2007). The loss of biodiversity is not limited to endangered species, although they undoubtedly attract more attention in terms of conservation efforts and use of resources (Ehrlich, 1994; Myers, 1996). This holds particularly true for species of high cultural importance for indigenous people, such as the caribou (*Rangifer tarandus*: Linnaeus, 1758), an indicator species for the entire boreal forest biome of North America (Vors & Boyce, 2009). For caribou of western Canada, the risks of biodiversity loss are clearly depicted in conservation practices at national and provincial levels, as some groups (designatable units, DU) and populations are listed as endangered, threatened or under concern (Species at Risk Act; COSEWIC 2011). Moreover, the complication of species protection increases significantly as caribou are comprised of a number of subspecies and numerous subpopulations with different life‐history strategies, some of which are in decline or at immediate risk, whilst others are locally extirpated (Festa‐Bianchet, Ray, Boutin, Côté, & Gunn, 2011; Wittmer, Mclellan, et al., 2005).

Whereas barren‐ground caribou are synonymous with long‐distance migrations of huge local populations across the arctic tundra, seasonal migratory behaviour also occurs in small populations at smaller scales in woodland caribou (Canadian Rockies, McDevitt et al., 2009; Ontario, Avgar, Mosser, Brown, & Fryxell, 2013). Local populations were previously identified as herds and included a more updated mapping, representing the full caribou range across Canada (Environment Canada 2011). Recent studies have shown that migratory woodland caribou have a unique life history in many populations (McDevitt et al., 2009; Weckworth, Musiani, McDevitt, Hebblewhite, & Mariani, 2012) exhibiting seasonal altitudinal migration (McDevitt et al., 2009), usually influenced by food availability and predation avoidance (Bischof et al., 2010; Hebblewhite & Merrill, 2007), in contrast to the stereotypical latitudinal migrations of barren‐ground caribou (Bergman, Schaefer, & Luttich, 2000; Musiani et al., 2007). Such movements are increased in rates and ranges during autumn (Ferguson & Elkie, 2004) by both sexes, a period that coincides with breeding season as females focus on reproduction and encounter with males (Fuller & Keith, 1981; Rettie & Messier, 2001). However, unlike their barren‐ground relatives, not all individuals display migratory behaviour. Most local populations are partially migratory (Chapman, Bronmark, Nilsson, & Hansson, 2011) where some individuals migrate and others remain sedentary as residents on their shared winter (or summer) ranges year‐round. Thus, partial migration may result in fine‐scale genetic structure within a local population of woodland caribou that may be impacted differently by human‐caused habitat fragmentation. Few studies have investigated the negative effects of human activity on the genetic connectivity of partially migratory populations.

Woodland caribou populations across Alberta and British Columbia (BC) have declined drastically. Current assessments indicate an approximate 50% loss of individuals every eight years in Alberta (Hervieux et al., 2013) and no long‐term viability of ten local populations in BC (Wittmer, Ahrens, & McLellan, 2010). These declines are attributed to habitat degradation and fragmentation largely due to natural resource extraction activities, which in turn increased wolf predation (Hervieux et al., 2013; Latham, Latham, Boyce, & Boutin, 2011; Polfus, Hebblewhite, & Heinemeyer, 2011; Wittmer, Sinclair, & McLellan, 2005). The increased incidental mortality by predators is attributed to “apparent competition” with moose or deer (normally the primary prey target for wolves) as a result of these habitat changes (DeCesare, Hebblewhite, Robinson, & Musiani, 2010; McLoughlin, Dzus, Wynes, & Boutin, 2003; Wittmer et al., 2010). There is concern that detrimental levels of predation and fragmentation have deleterious effects on population trends and genetic diversity, particularly of small and isolated local populations, further contributing to population declines. Genetic diversity, as determined by gene flow, stochastic genetic drift and/or selection, allows natural populations to adapt to local conditions (Gandon & Nuismer, 2009; North, Pennanen, Ovaskainen, & Laine, 2011). Genetic variation and gene flow are higher in large populations that typically accumulate more mutations than smaller ones (Star & Spencer, 2013). In vulnerable and small caribou populations, the consequences of drift and restricted gene flow are more profound as they are suspected to decrease genetic variation and enhance isolation, respectively (Serrouya et al., 2012; Weckworth et al., 2013).

Adaptive genetic variation is crucial to species conservation (Holderegger, Kamm, & Gugerli, 2006). Recovery plans need to be designed to reverse population declines and restore habitat in the short term and protect species gene pools in the long term, whilst accounting for spatial structure (Hice, Duffy, Munch, & Conover, 2012; McKay & Latta, 2002). It is important to monitor and map the geographic distribution of the variation requiring protection, particularly as adapted traits change across species ranges. Moreover, complexity increases in the presence of hybrid zones, where a mixture of characteristics is detected. In caribou, behavioural traits are documented to associated DUs, where seasonally migratory mountain caribou are genetically distinguished from the sedentary boreal type (McDevitt et al., 2009; Weckworth et al., 2012). Migratory individuals exhibit higher connectivity as they traverse a range of different landscapes, avoiding reproductive isolation. Conversely, sedentary animals cover a more restricted area and could potentially suffer more from habitat fragmentation.

Landscape genetic research has largely focused on evaluating biological processes at the group/population level. Individual‐based analyses are less common, despite having the ability to detect genetic discontinuities at a finer scale (Fontaine et al., 2007; Landguth et al., 2010; Zhu, Zhan, Meng, Zhang, & Mei, 2010). This approach exhibits an increased sampling coverage at landscape levels (Blair et al., 2012; Prunier et al., 2013), with at least double the magnitude of power for correlations between genetic and geographic distances when using simple Mantel tests (Legendre & Fortin, 2010). The use of individuals as discrete analytical units has proven to be more beneficial to group approaches regardless of the methodology applied (Luximon, Petit, & Broquet, 2014). Moreover, for species that exhibit varying regional migration patterns, such as the caribou of the Canadian Rockies (McDevitt et al., 2009), integrating high‐resolution molecular and ecological data allows for a better understanding of differentiation patterns that inform management and conservation measures (Storfer et al., 2007). Individual‐based landscape approaches are useful in small‐scale habitats fragmented by recent anthropogenic activities.

For caribou, the situation is compounded by complex local population dynamics related to severe fluctuations in population sizes and distributions (Fortin et al., 2013; Taillon, Fest‐Bianchet, & Côté, 2012). Despite stated short‐ and long‐term management objectives aimed at ensuring species survival, connectivity between local populations and evolutionary potential (Environment Canada 2014), there are continued declines across Alberta and British Columbia (Hebblewhite, White, & Musiani, 2010; Hervieux et al., 2013; Wittmer, Mclellan, et al., 2005). Current approaches fail to protect habitat within population range areas and overlook the importance of intermediary habitat, leading to further isolation of local populations. Therefore, connectivity should be assessed by a range of variables that include the geographic distance between individuals, habitat fragmentation and barriers (due to anthropogenic or climatic factors), and predation risk, as they are of primary concern for management.

In this study, we evaluated factors contributing to connectivity and isolation among local populations of threatened caribou of the Canadian Rockies. First, we quantified gene flow among geographically predefined local populations to identify those that likely export or receive the highest number of genetic migrants. This study explicitly addressed two different kinds of migration and their relationship: genetic migration among local populations (dispersal over ecological time scales) and behavioural migration in the form of seasonal migratory behaviour between seasonal ranges within an individual's home range. Second, we performed individual‐based analysis to examine how multiple topographic and environmental variables (natural and anthropogenic) affect genetic distance. Third, we used both Mantel and partial Mantel tests in a reciprocal causal modelling approach (Cushman, Wasserman, Landguth, & Shirk, 2013) to compare multiple competing models explaining genetic distance, highlighting the factors most sensitive to population isolation, and thus the most important to be managed for connectivity. Finally, caribou individuals exhibit contrasting migratory behaviours that can impact gene flow. We analysed separately individuals that exhibit seasonal migration and those that do not (McDevitt et al., 2009), taking advantage of the individual‐level behavioural data we had on spatial movement strategies for sampled caribou, to interpret their different contributions to gene flow in this threatened species.

## 2 Materials and Methods

### 2.1 Study area and individual samples

The 70,000‐km^2^ study area lies in the Central Rockies Ecosystem and includes montane, subalpine and alpine ecoregions with long winters and short, dry summers. The topography is comprised of flat valley bottoms surrounded by the Rocky Mountains (400–3937 m). The protected areas of Banff and Jasper national parks are located in the western mountainous region, whereas the higher human impact areas (roads, seismic exploration lines, forestry cut blocks, well pads and railways) occur predominantly in the eastern boreal foothill region.

**Figure 1:**
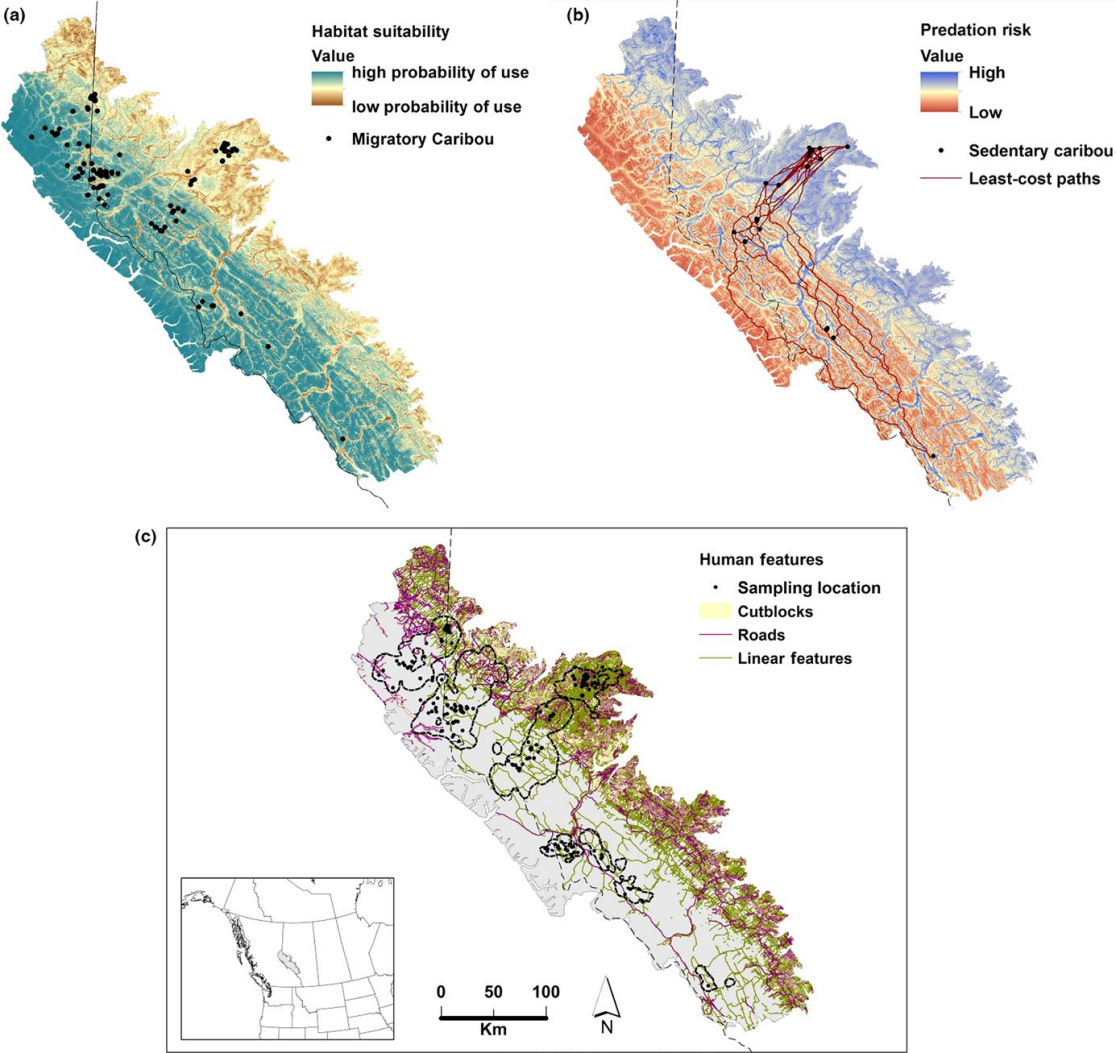
Maps depicting caribou resistance surfaces under a 30 m pixel resolution. These include the (a) habitat suitability, (b) predation risk from wolves with least‐cost paths of sedentary caribou and (c) human footprint features (roads, nonroad linear features and cutblocks) and sampling locations of individual caribou (207 specimens) in the Canadian Rocky Mountains, Alberta (AB) and British Columbia (BC) provinces

A total of 207 adult female caribou (147 global positioning system [GPS] collared and 60 noncollared) from eight different local populations in west‐central Alberta and eastern British Columbia, Canada (Figure 1c), were genotyped at 14 microsatellite loci as described by Weckworth et al. (2012, 2013). Sampling included individuals from all known local populations in the area. We focused on adult females as they produce and raise offspring alone, rendering them the most important element to population dynamics of polygynous ungulates (Gaillard, Festa‐Bianchet, & Yoccoz, 1998; Gaillard, Festa‐Bianchet, Yoccoz, Loison, & Toigo, 2000). Similarly, caribou landscape genetic studies have largely focused on females (Boulet, Couturier, Cote, Otto, & Bernatchez, 2007; McLoughlin, Paetkau, Duda, & Boutin, 2004), as no significant differences have been reported between sexes. We used previously developed nonlinear movement modelling methods (Bunnefeld et al., 2011) to classify migratory behaviour of all individual caribou (DeCesare et al., 2012). Briefly, individual caribou were defined as migratory or sedentary based on ungulate migration and their movement between seasonally nonoverlapping, allopatric ranges (Craighead, Atwell, & O'Gara, 1972). The overlap between summer ranges (1 July–15 September; Dyer, O'Neill, Wasel, & Boutin, 2001, 2002) and winter ranges (1 December–30 April; Smith, Ficht, Hobson, Sorensen, & Hervieux, 2000) for collared individuals was calculated. Caribou were considered migratory when showing nonoverlapping ranges, and sedentary when ranges overlapped seasonally (McDevitt et al., 2009).

### 2.2 Resistance surfaces

Analyses of connectivity were performed to identify corridors or barriers using caribou resource selection function models (RSF, Manly, McDonald, Thomas, McDonald, & Erickson, 2002; DeCesare et al., 2012) and assess the relative role of potential environmental, predation and anthropogenic drivers on genetic differentiation in caribou individuals. Adult female caribou GPS collar location data were combined with ecogeographic (i.e. topographic, climatic and vegetation) variables in a scale‐independent, used–available design (Manly et al., 2002) to estimate the relative probability of caribou use across scales (for details see DeCesare et al., 2012) on 30‐m spatial resolution layers (RSF; Figure 1a). Additionally, a layer of equal resolution for wolf‐predation risk was created (PRR; Figure 1b). The wolf risk model was developed based on the probabilities of encounter and predation of caribou by GPS‐collared grey wolves, *Canis lupus* (DeCesare, 2012), following the exclusion of anthropogenic features. The modelling of landscape resistance layers derived from these two inputs of caribou RSF and predation risk was described by DeCesare et al. (2012). Finally, anthropogenic footprints (forestry cut blocks, roads and other nonroad linear features) were also considered as factors impeding caribou dispersal (Figure 1c), as individuals are known to respond to such landscape fragmentation features (Apps & McLellan, 2006).

### 2.3 Landscape genetic analysis

#### 2.3.1 Local population‐based analysis

Directional estimates of contemporary gene flow between local populations were estimated with BayesAss 1.3 (Wilson & Rannala, 2003). BayesAss uses Markov chain Monte Carlo methods to estimate gene flow, does not assume Hardy–Weinberg or migration–drift equilibriums among populations and performs well with high genetic structure. We ran three replicates of 3 × 10^6^ MCMC iterations with sampling frequency of 2,000 and 10^6^ iterations burn‐in. Different delta values in allele frequencies (*p*), inbreeding coefficient (*F*) and migration rate (*m*) were tested and adjusted to numbers over a range of 40%–60% changes. We also determined 95% confidence intervals for migration rates. The output was divided into two matrices (*M*_1_ and *M*_2_), each representing one direction of contemporary gene flow between two local populations.

A nonparametric multivariate analysis of variance was used to determine the association between local population effective (*N*_e_) and census (*N*_c_) sizes as predictor factors, with pairwise genetic distance (*F*_ST_, Supporting Information Appendix S1) and two dispersal matrices (*M*_1_ and *M*_2_) as response matrices. Calculations of distance‐based multivariate analyses for linear models were performed in DISTLM5 (Anderson, 2004). Values of *N*_e_ were calculated as described by Weckworth et al. (2013) in LDNe, using a linkage disequilibrium method (Waples & Do, 2010). *N*_c_ values per local population were provided by Alberta Sustainable Resource Development and Alberta Conservation Association (2010). To evaluate the effect of local population size and evolutionary processes, such as genetic drift, on connectivity, average local population geographic distances (Euclidean distance between centre point of each local population home range) were used as a covariate for all three matrices (*F*_ST_, *M*_1_ and *M*_2_). Finally, *F*_ST_ was used as another covariate when analysing dispersal (*M*_1_ and *M*_2_). This allows us to also examine the effect of population size (effective or census) on gene flow, whilst correcting for the influence of drift. In theory, connectivity will be elevated among large populations, irrespective of distances, whereas smaller ones exhibit higher chances to remain genetically isolated, allowing drift to erode genetic variation.

#### 2.3.2 Individual‐based analysis

The genetic distance matrices, *a*_*r*_ (Rousset, 2000), among (i) all individuals, (ii) seasonally migratory and (iii) sedentary caribou were calculated in SPAGeDI 1.4 (Hardy & Vekemans, 2002). Moreover, three maximum‐likelihood estimates of relatedness among (i) all, (ii) migratory and (iii) sedentary caribou were calculated in ML‐Relate (Kalinowski, Wagner, & Taper, 2006). Values were translated as 0 unrelated, 0.0001–0.25 weakly related, 0.2501–0.5000 moderately related, 0.5001–0.9999 highly related and 1.0 fully related. This approach accommodates for null alleles on maximum‐likelihood estimates of relatedness between individuals of unknown ancestry, allowing the use of every locus available. Significance was tested using 5,000 randomizations of alleles among individuals. Overall, 126 migratory and 21 sedentary caribou were identified, with the remaining 60 unclassified (noncollared individuals) given a lack of telemetry data. Here, a relatively smaller sample size of sedentary compared to seasonal migratory caribou was used for the same geographic area to draw inference. However, this is the first study identifying differences between seasonally migratory and sedentary individuals of a protected species to our knowledge, and predictions even on relatively small sample sizes can be useful in guiding future research efforts (Wisz et al., 2008).

We tested how individual covariates affect genetic differentiation among individuals. We associated each sampled caribou with attributes that were grouped into (i) spatial (longitude and latitude of sample location), (ii) topographic (vegetation and elevation), (iii) environmental (snow cover) and (iv) local population characteristic variables (census population size, designatable unit (DU) and local population ID) (according to the local population in which they were sampled). The topographic and environmental variables were calculated based on the average home range (about 500 km^2^) of woodland caribou in Alberta (Stuart‐Smith, Bradshaw, Boutin, Hebert, & Rippin, 1997; Tracz, LaMontagne, Bayne, & Boutin, 2010). Vegetation was described by 15 classes of land cover type and normalized difference vegetation index (NDVI) (DeCesare et al., 2012); we reduced the dimensions of the data to two variables through principal component analysis (PCA) in SPSS 21. Vegetation classes were categorized using continuous and categorical mapping products from Landsat 5 or 7 Thematic Mapper (TM) sensors as described by McDermid (2006).

Marginal tests were run to assess the variation explained by each variable (longitude, latitude, snow cover, elevation, census population size of local population, DU, local population) or sets of variables (spatial coordinates, vegetation) when considered alone on genetic distance and relatedness matrices using DISTLM5 (Anderson, 2004). This comparison permits the evaluation of the variables’ effect on gene flow among individuals. Moreover, we performed conditional tests between the genetic or relatedness distances and the aforementioned predictors, with the individual coordinates (longitude and latitude) as covariates. This allowed an examination of the extent to which the predictors describe genetic diversification, aside from what is explained by geographic distance alone. In addition, the forward selection method in DISTLM *forward* (Anderson, 2003) was used to determine which sets of variables best modelled genetic variation and relatedness among all caribou, after examining any correlation between variables. This approach fits each variable sequentially (one at a time), whilst specifying the variance component described by each variable; we tested parameters including spatial coordinates, elevation, snow cover, vegetation, DU, census population size and local population. All *p* values were obtained after 9,999 permutations.

We tested multiple hypotheses of genetic differentiation, including absence of spatial structure, presence of anthropogenic barriers, predation risk and unsuitable habitat (assessed with RSFs that excluded predator or human factors). The null model of isolation by distance (IBD) was initially measured by calculating all logarithmic pairwise geographic Euclidean distances. Additionally, landscape resistance and wolf‐predation risk between the three groups of individuals (all, migratory and sedentary) were estimated using least‐cost path (LCP_RSF_ and LCP_PRR_, respectively) analyses on each resistance surface using the Landscape Genetics ArcToolbox (Etherington, 2011) in ArcGIS 10.0 (ESRI). Finally, influences of anthropogenic impact on gene flow (isolation by barrier, IBB) were examined by measuring the number of barriers (forestry cut blocks, roads and nonroad linear features, Figure 1c; IBB_Cutblocks_, IBB_Roads_ and IBB_LinearFeatures_) on a Euclidean line among all separate individual pairs.

The influence of spatial separation (IBD), habitat suitability (LCP_RSF_), predation risk (LCP_PRR_) and hypothesized human barriers to movement (IBB_Roads_, IBB_Cutblocks_ IBB_Linear Features_) on the genetic and relatedness distances among caribou was tested using simple Mantel tests (Smouse, Long, & Sokal, 1986) in ZT 1.1 (Bonnet & Van de Peer, 2002) under 100,000 permutations. We then used partial Mantel tests following the original causal modelling framework, which has a higher power to detect landscape influences on genetic structure in an individual‐based analysis (Cushman & Landguth, 2010; Cushman, McKelvey, Hayden, & Schwartz, 2006). We did this by controlling for the effects of geographic, landscape resistance, predation and barrier distances against the genetic (*a*_*r*_) and relatedness (R) matrices. Finally, we employed an improved version of the causal modelling approach (Cushman et al., 2013), to minimize problems of false positives (type I errors) due to spurious correlations found in partial Mantel tests. We evaluated multiple topographic, environmental and local population characteristic variables, isolation by distance, habitat suitability, predation risk and human barriers to identify potential drivers of gene flow and relatedness among caribou individuals. The reciprocal causal modelling approach is based on two partial Mantel tests for each combination of alternative hypotheses. The extent of difference between these two tests would define the supported hypothesis; the latter should have large positive values compared to all alternative models.

## 3 Results

### 3.1 Gene flow in caribou

The highest proportion of individuals originated from their own local population (Table 1). Bidirectional estimates of female gene flow (dispersal migration) among localities were low (*m *< 0.030) to moderate (0.030* *< *m *< 0.100). There were two cases where gene flow was high (0.100* *< *m*); these were from TQN to BRZ and RPC to NAR, with the highest value occurring from RPC to NAR (0.225). Overall, genetic migration rates between local population pairs were symmetric, with few cases of strong asymmetry; there were higher emigration rates from RPC to NAR and TQN to BRZ than vice versa (Table 1). Additionally, RPC showed the highest net emigration rate (the sum of outgoing minus the sum of incoming gene flow). The maximum‐likelihood estimates of relatedness between individuals of different local populations ranged from 0 to 0.500, whilst higher values were observed within each local population (from 0 to 0.960) with higher values being indicative of occurrence of half‐ and full‐siblings.

**Table 1:** Directional pairwise gene flow estimates per local population of caribou in west‐central Alberta and eastern British Columbia, Canada

From/To	ALP	BNP	BRZ	MAL	TQN	LSM	NAR	RPC
ALP	0.821 (0.735–0.899)	*0.017 (0*–*0.081)*	*0.022 (0*–*0.087)*	*0.018 (0*–*0.082)*	0.004 (0–0.027)	0.001 (0–0.011)	*0.008 (0*–*0.035)*	0.006 (0–0.033)
BNP	0.005 (0.000–0.024)	0.820 (0.724–0.940)	0.021 (0–0.91)	*0.019 (0*–*0.082)*	0.004 (0–0.026)	0.001 (0–0.011)	0.003 (0–0.018)	0.002 (0–0.010)
BRZ	0.004 (0.000–0.022)	***0.055 (0.014***–***0.137)***	0.712 (0.668–0.813)	0.019 (0–0.080)	0.004 (0–0.025)	0.001 (0–0.012)	0.003 (0–0.016)	0.002 (0–0.012)
MAL	0.013 (0.000–0.04)	0.016 (0–0.083)	*0.022 (0*–*0.087)*	0.792 (0.724–0.878)	0.082 (0.008–0.121)	0.001 (0–0.11)	0.003 (0–0.018)	0.002 (0–0.014)
TQN	***0.012 (0.001–0.069)***	*0.042 (0–0.079)*	***0.116 (0.036–0.221)***	*0.097 (0–0.132)*	0.883 (0.847–0.909)	0.002 (0–0.012)	0.003 (0–0.019)	0.002 (0–0.011)
LSM	***0.050 (0.015–0.103)***	*0.016 (0–0.078)*	*0.022 (0–0.083)*	*0.018 (0–0.082)*	*0.004 (0–0.028)*	0.989 (0.959–1.000)	*0.003 (0–0.018)*	0.002 (0–0.054)
NAR	0.007 (0–0.032)	*0.016 (0–0.076)*	*0.021 (0–0.087)*	*0.018 (0–0.082)*	*0.004 (0–0.025)*	0.002 (0–0.012)	0.752 (0.704–0.812)	0.014 (0–0.057)
RPC	***0.076 (0.013–0.184)***	*0.017 (0–0.078)*	*0.022 (0–0.087)*	*0.018 (0–0.081)*	*0.014 (0–0.101)*	0.002 (0–0.016)	***0.225 (0.160–0.279)***	0.971 (0.913‐0.999)

Underlined values are indicative of gene flow originating from their own local population. Values in brackets are the 95% confidence intervals. Values in italics represent directionally higher pairwise emigration rates; values in bold italics correspond to critically higher emigration rates between two areas. Local populations are defined as ALP, A La Peche; BNP, Banff National Park; BRZ, Brazeau; LSM, Little Smoky; MAL, Maligne; NAR, Narraway; RPC, Redrock‐Prairie Creek; TQN, Tonquin.

### 3.2 Landscape genetic analysis

#### 3.2.1 Local population level

The local population‐level distance‐based multivariate analysis showed that genetic distances (*F*_ST_) were significantly associated with effective population size only (*p *= .012), explaining 32.3% of the variation. Such an association would not be unexpected as both *F*_ST_ and *N*_e_ reflect genetic drift, and as it is unclear whether *N*_e_ has changed recently relative to the long‐term average. However, no influence of effective local population or census local population sizes was detected on *F*_ST_ values after controlling for geographic distance. Moreover, no significant correlation was detected among gene flow and effective or census local population sizes, before or after controlling for *F*_ST_.

#### 3.2.2 Individual level

All but three predictor factors (local population size, snow cover and vegetation) had a significant effect on genetic differentiation for the marginal tests, accounting for 2.00%–10.85% of its variation (Table 2). The smallest variation was explained by elevation and the largest portion by the spatial coordinates. When accounting for geographic distance, no single factor remained significantly associated with genetic differentiation among individual caribou (Table 2). With the forward selection procedure for a combined model of DISTLM, three variables explained 10.88% of the genetic variability, the foremost being the coordinates, whilst elevation and vegetation had minimal contributions (Table 2). These results remained the same despite the removal of snow cover variable, following multicollinearity analysis. The pairwise correlation coefficients among predictor factors were relatively low (Appendix S2). Elevation was correlated with snow cover (0.759). Additionally, relatedness variation was better explained by the local population of origin, followed by the spatial coordinates, with values ranging from 0.68% to 5.88% (marginal tests, Appendix S3). The conditional tests showed that none of the individual and sets of predictors could justify relatedness when geographic locality was included as a covariate. Consequently, in the sequential tests of the multiple regression model, none of the variables increased the sum of square values (<0.00001, Appendix S3).

**Table 2:** Effects of ten main predictor factors on genetic differentiation of 207 caribou

Predictor variables	Marginal tests			Conditional tests			Sequential tests		
	*F*	*p*	% var	*F*	*p*	% var	*F*	*p*	% var
*Nc*	15.92	.0001	7.21	2.90	.0963	1.26	–	–	–
Local population	0.97	.3314	3.79	−1.59	1.0000	−6.20	–	–	–
DU	4.46	.0181	2.13	−0.05	.9639	0.00	–	–	–
Latitude	20.35	.0001	9.03	NA	NA	NA	NA	NA	NA
Longitude	20.21	.0001	8.98	NA	NA	NA	NA	NA	NA
Coordinates	12.41	.0001	10.85	NA	NA	NA	12.45	.0001	10.85
Elevation	4.18	.0256	2.00	2.60	.1122	1.13	3.12	.0978	0.01
Snow cover	1.80	.3036	0.86	1.68	.1990	0.73	1.01	.4916	0.00
Vegetation	1.15	.4588	1.11	2.02	.1358	1.75	1.01	.1899	0.02

Variables were analysed individually (marginal), with spatial coordinates as covariables (conditional), and with a forward selection procedure for a combined model (sequential). *F* indicates test statistics, *p* shows probability values; %var represents the percentage of the genetic variation explained by each variable.

The Mantel results, testing associations between genetic and all other pairwise distance matrices among caribou individuals, exhibited positive and significant correlations (Table 3). The landscape resistance model (RSF) provided the best fit to genetic distance among all individuals (*r *= .25, *p *< .001), with values for sedentary individuals nearly three times higher (*r *= .47, *p *< .001) than in seasonally migratory caribou (*r *= .17, *p *< .001). Landscape resistance was followed by geographic distance and predation risk, whereas the amount of variation explained by anthropogenic barriers was negligible. All correlations between relatedness and pairwise distances were negative and statistically significant (Table 3).

**Table 3:** Correlations between gene flow/relatedness and geographic distance (IBD), least‐cost (LCP) and human made barriers (IBB) among all individual, sedentary and migratory caribou using simple Mantel’s tests

	Genetic distance (*a*_*r*_)		Relatedness (R)	
	Mantel's *r*	*p*	Mantel's *r*	*p*
IBD	.2399	.00001	−.2279	.00001
LCP_RSF_	.2488	.00001	−.2300	.00001
LCP_PRR_	.1899	.00001	−.2477	.00001
IBB_Roads_	.0990	.00006	−.1516	.00001
IBB_Cutblocks_	.0638	.00710	−.1208	.00001
IBB_LinearFeatures_	.0735	.00847	−.1484	.00001
IBD__Sedentary_	.4516	.00001	−.3636	.00002
LCP_RSF_Sedentary_	.4692	.00001	−.3546	.00001
LCP_PRR_Sedentary_	.4204	.00005	−.4188	.00001
IBD__Migratory_	.1532	.00001	−.1661	.00001
LCP_RSF_Migratory_	.1649	.00002	−.1674	.00001
LCP_PRR_Migratory_	.1066	.00216	−.1702	.00001

RSF, resource selection function model; PRR, wolf predation risk model. *r* is the correlation index of Mantel test; *p* shows probability values.

Causal modelling revealed significant associations between gene flow and habitat suitability when Euclidean distances were controlled for and vice versa, indicating that both Euclidean and resistance distances had an effect on genetic variability among all individuals (Appendix S4A). In contrast, significant correlations were found between genetic and Euclidean distances after the removal of predation and anthropogenic barrier matrices (Appendix S4A). Similarly, habitat suitability explained genetic differentiation of individual caribou (Appendix S4B). In analyses predicting the best fit of genetic relatedness, partial Mantel values were significant, indicating that all variables had an effect on relatedness (Appendix S5). Therefore, in addition to geographic distances and habitat suitability, predation risk and anthropogenic footprints significantly affected genetic relatedness.

In this study, 126 individuals were identified as being seasonally migratory. The amount of gene flow among seasonally migratory caribou was better explained by habitat suitability when geographic distance was included as a covariate. This signal was not detected for predation risk (Appendix S6A). The partial Mantel values for relatedness were significant for all variables (Appendix S6B). For the sedentary caribou, none of the resistance matrices could explain gene flow when the effect of log Euclidean distance was controlled (*p *< .05, Appendix S7A). Additionally, relatedness was better explained by Euclidean distance once the predation correlation was removed. The relationship between the relatedness matrix and predation risk was significant after accounting for landscape resistance (*r *= −.1971, *p *= .0022; Appendix S7B).

**Figure 2:**
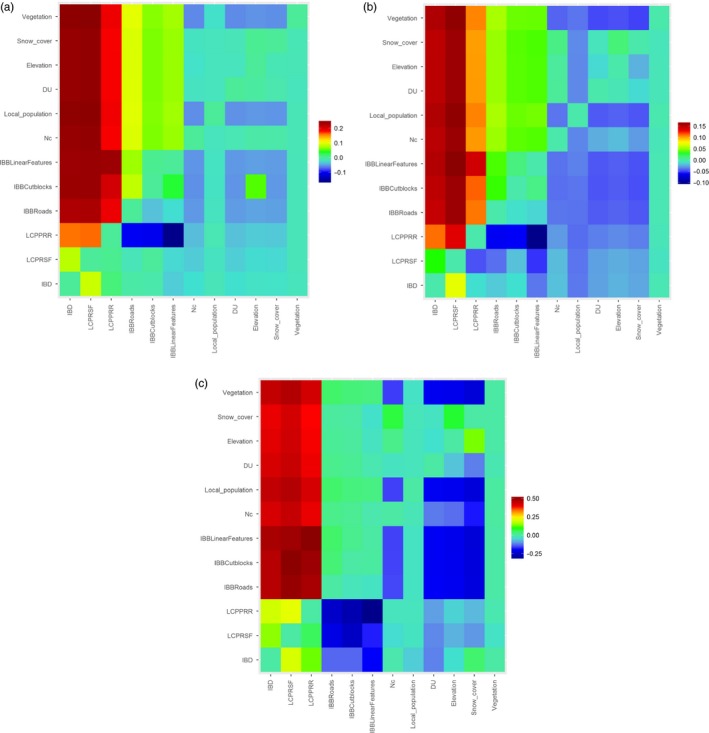
Confusion matrices of reciprocal causal modelling on caribou gene flow. These include the (a) complete caribou data set, (b) migratory and (c) sedentary individuals. Columns indicate principal models, whilst rows indicate alternative models. The colour gradient from blue to red indicates support for the principal model independent of the alternative model. A model that is fully supported should exhibit all positive values vertically, and negative values in the horizontal dimension. Nc is the local population census (*Nc*) sizes, and DU represents designatable units

We found that three models were strongly supported among caribou gene flow when using the method of reciprocal causal modelling. These were the Euclidean distance (IBD), habitat suitability (LCP_RSF_) and predation risk (LCP_PRR_) for all data sets (Figure 2). Both IBD and habitat suitability were the models with the highest support in the full caribou data set (Figure 2a), whereas habitat suitability and predation risk have higher support for the migratory and sedentary individuals, respectively (Figure 2b,c). The remaining models were not supported, as they exhibited small or negative values compared to the main three models. Conversely, all models were incapable of explaining relatedness, as they were not fully supported (Appendix Fig. S1).

## 4 Discussion

In this study, gene flow appeared restricted to adjacent local populations and was not affected by population sizes and seasonal migration, with most mating occurring among neighbours. This result is corroborated by telemetry data that showed most individual movements occurred within a local population's home range (McDevitt et al., 2009). Low to moderate connectivity among neighbouring local populations was documented, except for the Little Smoky local population, which remains isolated without any apparent immigration (Table 1). Even where land use and anthropogenic fragmentation occurs at lower levels (Figure 1c), gene flow was limited to distances less than 100 km; for example, values were higher from RPC and TNQ towards NAR and BRZ, respectively (Table 1). Members of each local population are still more likely to breed within their same local population, potentially leading to high levels of inbreeding due to the absence of genetic exchange with other local populations. Although caribou are highly mobile, limited migration may also be observed in species with long‐range dispersal capabilities (Hull, Hull, Sacks, Smith, & Ernest, 2008).

We integrated evolutionary and ecological approaches to better understand the relationships between genetic structure and gene flow with topographic, climatic and vegetation predictors of caribou natural history (DeCesare et al., 2012). We found significant correlations between genetic distances and sampling locality, local population size, DU and elevation (in order of explaining most variance, Table 2). Positive and significant correlations between genetic distances and spatial coordinates could be indicative of an average increase in genetic differentiation from south‐west to north‐east. Interestingly, the most genetically distinct local population documented is the Little Smoky population, found in the easternmost sampling locality of the study area (McDevitt et al., 2009; Weckworth et al., 2012). However, this directional pattern is unlikely to be a simple result of population geographic location. Our findings support the hypothesis that genetic differentiation among caribou individuals is influenced separately by several ecological as well as spatial variables (Table 2). Moreover, vegetation availability, elevation and weather conditions were also used to evaluate connectivity due to foraging, migration to higher ground for predator avoidance and snow cover during winter, respectively. These are factors known to influence seasonal habitat selection (Bergerud, Ferguson, & Butler, 1990; Simpson, Terry, & Hamilton, 1997), especially in ungulates that undertake partial (Barnowe‐Meyer et al., 2010; Hebblewhite & Merrill, 2007; Plumb, White, Coughenour, & Wallen, 2009) and long‐distance seasonal migrations (Yannic et al., 2014) in North America. Such influence is also reflected on population structure of predators that specialize on ungulate species, particularly wolves (Carmichael et al., 2007). However, after controlling for location, no single characteristic explained genetic variation (Table 2). Considering the regional migratory behaviour of the Rockies’ caribou (McDevitt et al., 2009), these results may be indicative of isolated local populations with limited gene flow from a small number of dispersers.

The evaluation of space, landscape and predation on genetic distances and relatedness revealed that habitat suitability and Euclidean distances both influenced genetic connectivity (Table 3), suggesting a similar effect of ecogeographic variables on gene flow. The impact of these variables on dispersal behaviour significantly affects connectivity among sedentary individuals, as evidenced by a threefold difference in structure over seasonal migrants (i.e. IBD__Migratory_ *r *= .1532, IBD__Sedentery_ *r *= .4516; Table 3). Conversely, migrants were less constrained by habitat resistance (Table 3, Figure 1). Migratory caribou demonstrated greater vagility and flexibility in habitat use, especially during their seasonal migration. Thus, habitat selection by caribou is influenced by environmental and habitat parameters (Bergerud, 1978) that vary among different DUs of woodland caribou (Jones, Gillingham, Seip, & Heard, 2007). However, habitat selection conditions might be more complex from those that promote gene flow, surpassing even those of predation risk.

Our results showed that IBD alone was not sufficient to explain caribou gene flow (Figure 2a). Habitat suitability followed by predation risk was also associated with overall gene flow (Figure 2a). Similarly, RSF‐based models have been shown to improve inference on connectivity, compared to simple IBD models, also in mountain goats (Shafer et al., 2012), suggesting that habitat selection is a good predictor of gene flow for ungulates. Following recent studies (Castillo, Epps, Davis, & Cushman, 2014; Cushman, Max, Whitham, & Allan, 2014), the reciprocal causal modelling approach could better identify supported values and strengthen the results and ranking to those based on simple or partial Mantel tests. The same three models were also supported in both migratory and sedentary data set, with a difference in the support level. For the migratory data set, habitat suitability showed higher support values, followed by the IBD and predation avoidance models (Figure 2b). For sedentary caribou, all models exhibited similar support values (Figure 2c). Such differences may reflect that the individuals analysed have different responses to geographic distances, habitat and particularly to predation (Middleton et al., 2013). Caribou populations are susceptible to decline via predation through increased adult mortality and depleted recruitment (Bergerud & Ballard, 1988; McLoughlin et al., 2003; Pinard, Dussault, Ouellet, Fortin, & Courtois, 2012). Although most woodland caribou populations are in danger of extinction, of particular risk are those exhibiting sedentary behaviour (Hervieux et al., 2013; McDevitt et al., 2009), as resident individuals are subjected to constant predation pressure. Conversely, migratory individuals avoid predators through seasonal spatial movements, and thus, wolf predation has a smaller effect on genetic variation compared to geographic distances and habitat suitability.

Despite evidence of caribou avoidance for anthropogenic barriers (DeCesare et al., 2012; Dyer et al., 2001; Fortin et al., 2013), the latter did not appear to influence gene flow after controlling for geographic distance. Anthropogenic features are known to decrease local population size (van Oort, McLellan, & Serrouya, 2011) and thus indirectly impact genetic variation, particularly in small local populations. However, here, there were no measurable statistical impacts on gene flow. These contradictory results may simply be a product of common molecular markers being largely insufficient when trying to resolve questions related to historically recent landscape alteration (Anderson et al., 2010). Similarly, inconclusive results on gene flow were also found in the prairie rattlesnake, *Crotalus viridis* (Weyer, Jørgensen, Schmitt, Maxwell, & Anderson, 2014), suggesting that connectivity is not always clear to detect when contemporary landscape fragmentation is accounted for. Additionally, our study area is small in context to the impact of these landscape changes, and our relatively homogenized environment has not reached the threshold at which significant impacts on gene flow can be detected.

The complexity of uniform management decisions in small areas is emphasized with the separation of caribou into migratory and sedentary animals. The role of individual caribou, or group of individuals, sharing life strategies is ignored and not incorporated into the species’ MU designation. This methodology relies on evaluating the role of each individual within a local population, as interindividual variation can have important consequences for dispersal and gene flow (McDevitt et al., 2013). The current assignment of individuals into populations may be flawed as they disregard individual variation in habitat selection. The support differences and inconsistencies of habitat selection between migratory and sedentary caribou were depicted by the different effects of landscape resistance on gene flow and relatedness (reciprocal causal modelling and partial Mantel tests, respectively). In migratory ungulates, movements are made in response to changes in food availability, habitat or weather (Bischof et al., 2010; Hebblewhite & Merrill, 2007); it is then expected that habitat suitability would be a better predictor of genetic differentiation than Euclidean distances or predation risk. Our findings lend further support to this hypothesis. Additionally, our results are based on females, suggesting that this gender is highly susceptible to alterations in landscape features (Yannic et al., 2014). No significant differences between female and male caribou were detected from both telemetry and genetic data (Boulet et al., 2007). Moreover, wolf‐predation avoidance does not seem to affect connectivity of seasonally migratory individuals as strong as habitat suitability and geographic distances (Figure 2b), as each caribou could be subjected to different local differences in predation risk (Bastille‐Rousseau et al., 2015). However, other predator–prey interactions, besides wolves, should be tested. Conversely, the genetic distances of sedentary female caribou are related to IBD (partial Mantel tests) and show that gene flow is spatially restricted. Less vagile individuals do not seem to move randomly across their distribution to avoid possible encounters with predators, and choose to stay closer to individuals of the same group.

Our study has clear management implications, as exemplified by our findings regarding the Little Smoky local population. In west‐central Alberta, this local population is at risk of extirpation (COSEWIC 2002) with restricted immigration, whilst outgoing gene flow is higher and directed mainly to the neighbouring local population of A La Peche. Overall connectivity was not measurably affected by anthropogenic barriers. Isolation by distance, followed by habitat suitability, and costs of movement across the local population's geographic range are the greatest contributors to connectivity. The Little Smoky animals are genetically distinct, exhibiting low levels of diversity, and limited spatial dispersal compared to other local populations (McDevitt et al., 2009; Weckworth et al., 2012), all consistent with an isolated, small boreal population (Hervieux et al., 2013) that is at risk of declining genetic diversity and inbreeding. The preservation of Little Smoky caribou, and the incurred costs, is a topic of considerable debate with regard to prioritizing conservation strategies towards local populations of nonimmediate risk of extirpation (Schneider, Hauer, Dawe, Adamowicz, & Boutin, 2012). There are clear advantages of individual translocation in endangered ungulates, particularly for small and isolated local populations (Balakrishnan, Monfort, Gaur, Singh, & Sorenson, 2003; Stephen et al., 2005). However, previous recovery efforts lacked adequate results for caribou and have never incorporated genetic data. We believe that if efforts to maintain the Little Smoky local population, and similarly small and isolated populations, continue, it is imperative that accurate information on gene flow is incorporated into management plans (Trumbo, Spear, Baumsteiger, & Storfer, 2013), particularly as alternative strategies of individual reintroductions have proven to be ineffective (Bergerud & Mercer, 1989; St‐Laurent & Dussault, 2012). Additionally, there is a continuous change in land use and its influence on ecological processes and biodiversity is poorly understood. Biotic resources are threatened by the rapid development of landscape, particularly in North America (Hansen et al., 2002; Travis, Theobald, & Fagre, 2002). Moreover, climate change poses new challenges to landscape and subsequently to biodiversity conservation. For caribou, habitat alterations will have serious consequences on connectivity. Gene flow has been significantly associated with habitat suitability, particularly for migratory individuals (Figure 2). Furthermore, migration is restricted to neighbouring areas (Table 1). Therefore, potential deteriorations of landscape and connectivity corridors among local population, particularly those found in protected areas, would result in the complete isolation of vulnerable local populations.

Our findings provide guidelines to caribou managers on the importance of incorporating genetic connectivity and ecological characteristics, such as migratory behaviour, into caribou management planning (Trumbo et al., 2013). Here, we used RSF models and found that geographic distances, habitat suitability and predatory risk can influence gene flow of individual female caribou across their ranges, whereas the level of resistance depends on whether an animal is sedentary or seasonally migratory. Even within caribou of the same local population, animals can have contrasting migratory patterns with significant differences in connectivity and habitat use. Effective conservation measures should consider individual habitat preferences to ensure long‐term viability for animals that are prone to seasonal movements across diverse areas. Furthermore, conservation management should not overlook demographic units of smaller distributional ranges, as connectivity among nonvagile individuals is more susceptible to the landscape impacts and predation. Therefore, viable decisions should be based on both large and more refined scales, whilst focusing on behaviour‐specific mitigation measures. A failure to detect processes influencing genetic connectivity and relatedness will have serious implications in conservation of caribou in the Canadian Rockies.
