# Supplementary material for: Environmental and anthropogenic drivers of connectivity patterns: A basis for prioritizing conservation efforts for threatened populations
Source: Evol Appl. 2016 Dec 20;10(2):199–211. doi: 10.1111/eva.12443 (PMC5253427; doi:10.1111/eva.12443)

**Supplementary Appendix 1:** Microsatellite pairwise differentiation among eight local populations used in this study. Local populations are defined as ALP: A La Peche, BNP: Banff National Park, BRZ: Brazeau, MAL: Maligne, TQN: Tonquin, LSM: Little Smoky, NAR: Narraway, RPC: Redrock-Prairie Creek. Values in brackets correspond to the number of samples per local population.

|  | **ALP** | **BNP** | **BRZ** | **MAL** | **TQN** | **LSM** | **NAR** | **RPC** |
| --- | --- | --- | --- | --- | --- | --- | --- | --- |
| **ALP (34)** | - |  |  |  |  |  |  |  |
| **BNP (5)** | 0.014 | - |  |  |  |  |  |  |
| **BRZ (6)** | 0.002 | 0.003 | - |  |  |  |  |  |
| **MAL (5)** | 0.002 | 0.003 | 0.002 | - |  |  |  |  |
| **TQN (18)** | 0.002 | 0.003 | 0.001 | 0.082 | - |  |  |  |
| **LSM (38)** | 0.002 | 0.003 | 0.001 | 0.004 | 0.019 | *-* |  |  |
| **NAR (46)** | 0.002 | 0.003 | 0.001 | 0.004 | 0.019 | 0.021 | - |  |
| **RPC (55)** | 0.006 | 0.008 | 0.001 | 0.004 | 0.018 | 0.022 | 0.017 | - |

**Supplementary Appendix 2:** Pairwise correlation coefficients among caribou gene flow variables.

|  | **Lon** | **Lat** | **Ne** | **Nc** | **Elevation** | **Snow Cover** | **Veg1** | **Veg2** | **DU** | **Herd** |
| --- | --- | --- | --- | --- | --- | --- | --- | --- | --- | --- |
| **Lon** | 1.000 | -0.620 | -0.829 | -0.646 | 0.160 | -0.170 | 0.076 | 0.047 | -0.484 | -0.582 |
| **Lat** | -0.620 | 1.000 | 0.572 | 0.338 | -0.748 | -0.333 | -0.297 | 0.016 | -0.272 | 0.502 |
| **Ne** | -0.829 | 0.572 | 1.000 | 0.552 | -0.245 | -0.036 | -0.047 | -0.065 | 0.326 | 0.567 |
| **Nc** | -0.646 | 0.338 | 0.552 | 1.000 | 0.104 | 0.317 | -0.016 | -0.145 | 0.335 | 0.412 |
| **Elevation** | 0.160 | -0.748 | -0.245 | 0.104 | 1.000 | 0.759 | 0.310 | -0.112 | 0.548 | -0.384 |
| **Snow Cover** | -0.170 | -0.333 | -0.036 | 0.317 | 0.759 | 1.000 | 0.155 | -0.042 | 0.434 | -0.065 |
| **Veg1** | 0.076 | -0.297 | -0.047 | -0.016 | 0.310 | 0.155 | 1.000 | 0.163 | 0.208 | -0.129 |
| **Veg2** | 0.047 | 0.016 | -0.065 | -0.145 | -0.112 | -0.042 | 0.163 | 1.000 | -0.085 | 0.053 |
| **DU** | -0.484 | -0.272 | 0.326 | 0.335 | 0.548 | 0.434 | 0.208 | -0.085 | 1.000 | -0.074 |
| **Herd** | -0.582 | 0.502 | 0.567 | 0.412 | -0.384 | -0.065 | -0.129 | 0.053 | -0.074 | 1.000 |

**Supplementary Appendix 3**: Effects of ten main predictor factors on relatedness among 207 caribou. Variables were analyzed individually (marginal), with spatial coordinates as covariables (conditional), and with a forward selection procedure for a combined model (sequential). F indicates test statistics, *p* shows probability values; %var represents the percentage of the genetic variation explained by each variable.

|  | **Marginal tests** | | | **Conditional tests** | | | **Sequential tests** | | |
| --- | --- | --- | --- | --- | --- | --- | --- | --- | --- |
| **Predictor Variables** | F | *p* | % var | F | *p* | % var | F | *p* | % var |
| Nc | 2.13 | 0.0001 | 1.03 | 1.73 | 0.1757 | 0.82 | - | - | - |
| Local Population | 1.55 | 0.0001 | 5.88 | 1.40 | 0.0656 | 5.28 | - | - | - |
| Ecotype | 2.67 | 0.0001 | 1.29 | 1.54 | 0.2150 | 0.73 | - | - | - |
| Latitude | 2.50 | 0.0001 | 1.21 | NA | NA | NA | NA | NA | NA |
| Longitude | 2.46 | 0.0001 | 1.19 | NA | NA | NA | NA | NA | NA |
| Coordinates | 2.50 | 0.0001 | 2.39 | NA | NA | NA | - | - | - |
| Elevation | 2.07 | 0.0001 | 1.00 | 1.25 | 0.2833 | 0.60 | - | - | - |
| Snow coverage | 1.40 | 0.0001 | 0.68 | 1.13 | 0.3223 | 0.54 | - | - | - |
| Vegetation | 1.44 | 0.0036 | 1.11 | 1.10 | 0.3637 | 1.05 | - | - | - |

**Supplementary Appendix 4**: Results of causal modeling with reported Mantel’s r and P values between Rousset's genetic and six distance matrices (Isolation by Distance, IBD; Habitat Suitability, LCP_RSF_; Predator avoidance, LCP_PRR_; Human footprint – Roads, IBB_Roads_; Human footprint – Cutblocks, IBB_Cutblocks_; Human footprint – Linear Features, IBB_LinearFeatures_) among 207 caribou, based on Partial Mantel tests after controlling for the Euclidean distance (A) and the least cost habitat suitability resistance model (B).

**A**

| (a) Habitat Suitability (LCP_RSF_) | | | | |  | | | |
| --- | --- | --- | --- | --- | --- | --- | --- | --- |
| LCP_RSF_ | IBD | |  | IBD | LCP_RSF_ | | |  |  |  |
| Partial r | P |  | Partial r | P | |  | Causal model supported | |
| 0.0886 | 0.0018 |  | 0.0820 | >0.0001 | |  | BOTH | |
|  |  |  |  |  | |  |  | |
| (b) Predator avoidance (LCP_PRR_) | | | | |  | | | |
| LCP_PRR_ | IBD | |  | IBD | LCP_PRR_ | | |  |  |  |
| Partial r | P |  | Partial r | P | |  | Causal model supported | |
| 0.0173 | 0.3313 |  | 0.1609 | >0.0001 | |  | Isolation by Distance | |
|  |  |  |  |  | |  |  | |
| (c) Human footprint – Roads (IBB_Roads_) | | | | |  | | | |
| IBB_Roads_ | IBD | |  | IBD | IBB_Roads_ | | |  |  |  |
| Partial r | P |  | Partial r | P | |  | Causal model supported | |
| -0.0060 | 0.4179 |  | 0.2271 | >0.0001 | |  | Isolation by Distance | |
|  |  |  |  |  | |  |  | |
| (d) Human footprint – Cutblocks (IBB_Cutblocks_) | | | | |  | | | |
| IBB_Cutblocks_ | IBD | |  | IBD | IBB_Cutblocks_ | | |  |  |  |
| Partial r | P |  | Partial r | P | |  | Causal model supported | |
| -0.0082 | 0.3826 |  | 0.2389 | >0.0001 | |  | Isolation by Distance | |
|  |  |  |  |  | |  |  | |
| (d) Human footprint – Linear Features (IBB_LinearFeatures_) | | | | |  | | | |
| IBB_LinearFeatures_ | IBD | |  | IBD | IBB_LinearFeatures_ | | |  |  |  |
| Partial r | P |  | Partial r | P | |  | Causal model supported | |
| -0.0324 | 0.1648 |  | 0.2382 | >0.0001 | |  | Isolation by Distance | |

**B**

| (a) Predator avoidance (LCP_PRR_) | | | | |  | | | |
| --- | --- | --- | --- | --- | --- | --- | --- | --- |
| LCP_PRR_ | control LCP_RSF_ | |  | LCP_RSF_ | LCP_PRR_ | | |  |  |  |
| Partial r | P |  | Partial r | P | |  | Causal model supported | |
| 0.0034 | 0.4548 |  | 0.1638 | >0.0001 | |  | Habitat Suitability | |
|  |  |  |  |  | |  |  | |
| (b) Human footprint – Roads (IBB_Roads_) | | | | |  | | | |
| IBB_Roads_ | LCP_RSF_ | |  | LCP_RSF_ | IBB_Roads_ | | |  |  |  |
| Partial r | P |  | Partial r | P | |  | Causal model supported | |
| -0.0141 | 0.3078 |  | 0.2298 | >0.0001 | |  | Habitat Suitability | |
|  |  |  |  |  | |  |  | |
| (c) Human footprint – Cutblocks (IBB_Cutblocks_) | | | | |  | | | |
| IBB_Cutblocks_ | LCP_RSF_ | |  | LCP_RSF_ | IBB_Cutblocks_ | | |  |  |  |
| Partial r | P |  | Partial r | P | |  | Causal model supported | |
| -0.0010 | 0.4873 |  | 0.2410 | >0.0001 | |  | Habitat Suitability | |
|  |  |  |  |  | |  |  | |
| (d) Human footprint – Linear Features (IBB_LinearFeatures_) | | | | |  | | | |
| IBB_LinearFeatures_ | LCP_RSF_ | |  | LCP_RSF_ | IBB_LinearFeatures_ | | |  |  |  |
| Partial r | P |  | Partial r | P | |  | Causal model supported | |
| -0.0282 | 0.2004 |  | 0.2400 | >0.0001 | |  | Habitat Suitability | |
|  |  |  |  |  | |  |  | |

**Supplementary Appendix 5**: Results of causal modeling with reported Mantel’s r and P values between relatedness and six distance matrices (Isolation by Distance, IBD; Habitat Suitability, LCP_RSF_; Predator avoidance, LCP_PRR_; Human footprint – Roads, IBB_Roads_; Human footprint – Cutblocks, IBB_Cutblocks_; Human footprint – Linear Features, IBB_LinearFeatures_) among 207 caribou, based on Partial Mantel tests after controlling for the Euclidean distance (A) and least cost habitat suitability resistance model (B).

**A**

| (a) Habitat suitability (LCP_RSF_) | | | | |  | | | |
| --- | --- | --- | --- | --- | --- | --- | --- | --- |
| LCP_RSF_ | IBD | |  | IBD | LCP_RSF_ | | |  |  |  |
| Partial r | P |  | Partial r | P | |  | Causal model supported | |
| 0.0293 | 0.0080 |  | -0.2133 | >0.0001 | |  | BOTH | |
|  |  |  |  |  | |  |  | |
| (b) Predator avoidance (LCP_PRR_) | | | | |  | | | |
| LCP_PRR_ | IBD | |  | IBD | LCP_PRR_ | | |  |  |  |
| Partial r | P |  | Partial r | P | |  | Causal model supported | |
| -0.0332 | 0.0028 |  | -0.1926 | >0.0001 | |  | BOTH | |
|  |  |  |  |  | |  |  | |
| (c) Human footprint – Roads (IBB_Roads_) | | | | |  | | | |
| IBB_Roads_ | IBD | |  | IBD | IBB_Roads_ | | |  |  |  |
| Partial r | P |  | Partial r | P | |  | Causal model supported | |
| -0.0246 | 0.0132 |  | -0.2728 | >0.0001 | |  | BOTH | |
|  |  |  |  |  | |  |  | |
| (d) Human footprint – Cutblocks (IBB_Cutblocks_) | | | | |  | | | |
| IBB_Cutblocks_ | IBD | |  | IBD | IBB_Cutblocks_ | | |  |  |  |
| Partial r | P |  | Partial r | P | |  | Causal model supported | |
| -0.0345 | 0.0007 |  | -0.2879 | 0.00001 | |  | BOTH | |
|  |  |  |  |  | |  |  | |
| (d) Human footprint – Linear Features (IBB_LinearFeatures_) | | | | |  | | | |
| IBB_LinearFeatures_ | IBD | |  | IBD | IBB_LinearFeatures_ | | |  |  |  |
| Partial r | P |  | Partial r | P | |  | Causal model supported | |
| -0.024 | 0.0269 |  | -0.2745 | >0.0001 | |  | BOTH | |

**B**

| (a) Predator avoidance (LCP_PRR_) | | | | |  | | | |
| --- | --- | --- | --- | --- | --- | --- | --- | --- |
| LCP_PRR_ | control LCP_RSF_ | |  | LCP_RSF_ | LCP_PRR_ | | |  |  |  |
| Partial r | P |  | Partial r | P | |  | Causal model supported | |
| -0.1161 | >0.0001 |  | -0.0677 | >0.0001 | |  | BOTH | |
|  |  |  |  |  | |  |  | |
| (b) Human footprint – Roads (IBB_Roads_) | | | | |  | | | |
| IBB_Roads_ | LCP_RSF_ | |  | LCP_RSF_ | IBB_Roads_ | | |  |  |  |
| Partial r | P |  | Partial r | P | |  | Causal model supported | |
| -0.0561 | >0.0001 |  | -0.1834 | >0.0001 | |  | BOTH | |
|  |  |  |  |  | |  |  | |
| (c) Human footprint – Cutblocks (IBB_Cutblocks_) | | | | |  | | | |
| IBB_Cutblocks_ | LCP_RSF_ | |  | LCP_RSF_ | IBB_Cutblocks_ | | |  |  |  |
| Partial r | P |  | Partial r | P | |  | Causal model supported | |
| -0.0649 | >0.0001 |  | -0.2071 | >0.0001 | |  | BOTH | |
|  |  |  |  |  | |  |  | |
| (d) Human footprint – Linear Features (IBB_LinearFeatures_) | | | | |  | | | |
| IBB_LinearFeatures_ | LCP_RSF_ | |  | LCP_RSF_ | IBB_LinearFeatures_ | | |  |  |  |
| Partial r | P |  | Partial r | P | |  | Causal model supported | |
| -0.0642 | >0.0001 |  | -0.1885 | >0.0001 | |  | BOTH | |
|  |  |  |  |  | |  |  | |

**Supplementary Appendix 6**: Results of causal modeling with reported Mantel’s r and P values for Rousset's genetic distance (A), relatedness (B) and three distance matrices (Isolation by Distance, IBD; Habitat Suitability, LCP_RSF_; Predator avoidance, LCP_PRR_) among migratory caribou (126 individuals), based on Partial Mantel tests.

**A**

| (a) Habitat suitability (LCP_RSF_) | | | | |  | | | |
| --- | --- | --- | --- | --- | --- | --- | --- | --- |
| LCP_RSF_ | IBD | |  | IBD | LCP_RSF_ | | |  |  |  |
| Partial r | P |  | Partial r | P | |  | Causal model supported | |
| 0.0706 | 0.0340 |  | 0.0343 | 0.0793 | |  | Habitat suitability | |
|  |  |  |  |  | |  |  | |
| (b) Predator avoidance (LCP_PRR_) | | | | |  | | | |
| LCP_PRR_ | IBD | |  | IBD | LCP_PRR_ | | |  |  |  |
| Partial r | P |  | Partial r | P | |  | Causal model supported | |
| -0.0112 | 0.3936 |  | 0.1112 | 0.0001 | |  | Isolation by Distance | |

**B**

| (a) Habitat suitability (LCP_RSF_) | | | | |  | | | |
| --- | --- | --- | --- | --- | --- | --- | --- | --- |
| LCP_RSF_ | control IBD | |  | IBD | LCP_RSF_ | | |  |  |  |
| Partial r | P |  | Partial r | P | |  | Causal model supported | |
| -0.0166 | 0.1134 |  | -0.1033 | >0.0001 | |  | Isolation by Distance | |
|  |  |  |  |  | |  |  | |
| (b) Predator avoidance (LCP_PRR_) | | | | |  | | | |
| LCP_PRR_ | IBD | |  | IBD | LCP_PRR_ | | |  |  |  |
| Partial r | P |  | Partial r | P | |  | Causal model supported | |
| -0.0379 | 0.0039 |  | -0.1043 | >0.0001 | |  | BOTH | |

**Supplementary Appendix 7**: Results of causal modeling with reported Mantel’s r and P values for Rousset's genetic distance (A), relatedness (B) and three distance matrices (Isolation by Distance, IBD; Habitat Suitability, LCP_RSF_; Predator avoidance, LCP_PRR_) among sedentary caribou (21 individuals), based on Partial Mantel tests.

**A**

| (a) Habitat suitability (LCP_RSF_) | | | |  | | | | |
| --- | --- | --- | --- | --- | --- | --- | --- | --- |
| LCP_RSF_ | IBD | |  | IBD | LCP_RSF_ | | |  |  |  |
| Partial r | P |  | Partial r | | P |  | Causal model supported | |
| 0.2031 | 0.0642 |  | 0.1458 | | 0.0379 |  | Isolation by Distance | |
|  |  |  |  | |  |  |  | |
| (b) Predator avoidance (LCP_PRR_) | | | |  | | | | |
| LCP_PRR_ | IBD | |  | IBD | LCP_PRR_ | | |  |  |  |
| Partial r | P |  | Partial r | | P |  | Causal model supported | |
| 0.1256 | 0.0771 |  | 0.1957 | | 0.0119 |  | Isolation by Distance | |

**B**

| (a) Habitat suitability (LCP_RSF_) | | | | |  | | | |
| --- | --- | --- | --- | --- | --- | --- | --- | --- |
| LCP_RSF_ | IBD | |  | IBD | LCP_RSF_ | | |  |  |  |
| Partial r | P |  | Partial r | P | |  | Causal model supported | |
| -0.0844 | 0.1065 |  | -0.1833 | 0.0111 | |  | Isolation by Distance | |
|  |  |  |  |  | |  |  | |
| (b) Predator avoidance (LCP_PRR_) | | | | |  | | | |
| LCP_PRR_ | IBD | |  | IBD | LCP_PRR_ | | |  |  |  |
| Partial r | P |  | Partial r | P | |  | Causal model supported | |
| -0.1971 | 0.0022 |  | -0.0900 | 0.0980 | |  | Predator avoidance | |

**Supplementary Figure 1**: Confusion matrices of reciprocal causal modeling on caribou relatedness. These include the: a) complete caribou dataset, b) migratory, and c) sedentary individuals. Columns indicate principal models, whilst rows indicate alternative models. The colour gradient from blue to red indicates support for the principal model independent of the alternative model. A model that is fully supported should exhibit all positive values vertically, and negative values in the horizontal dimension. Nc is the local population census sizes; and DU represents Designatable Units.


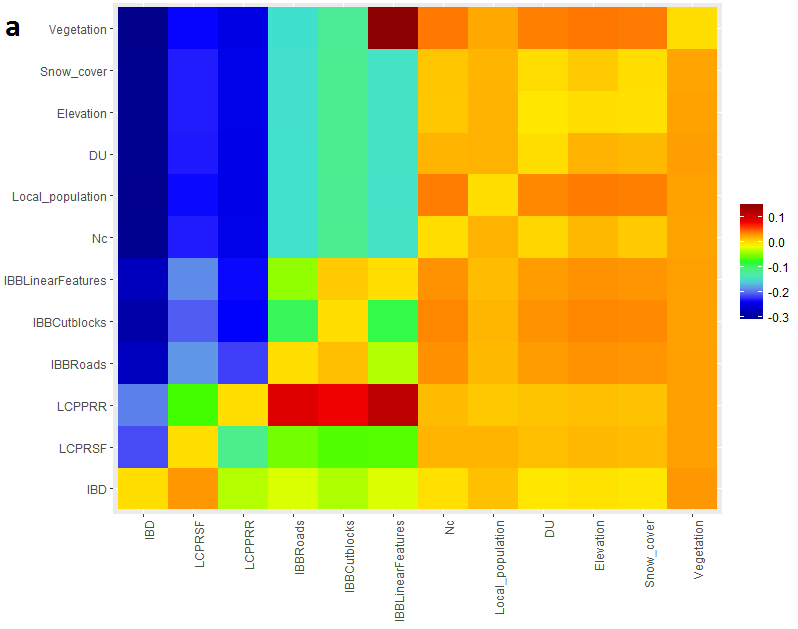


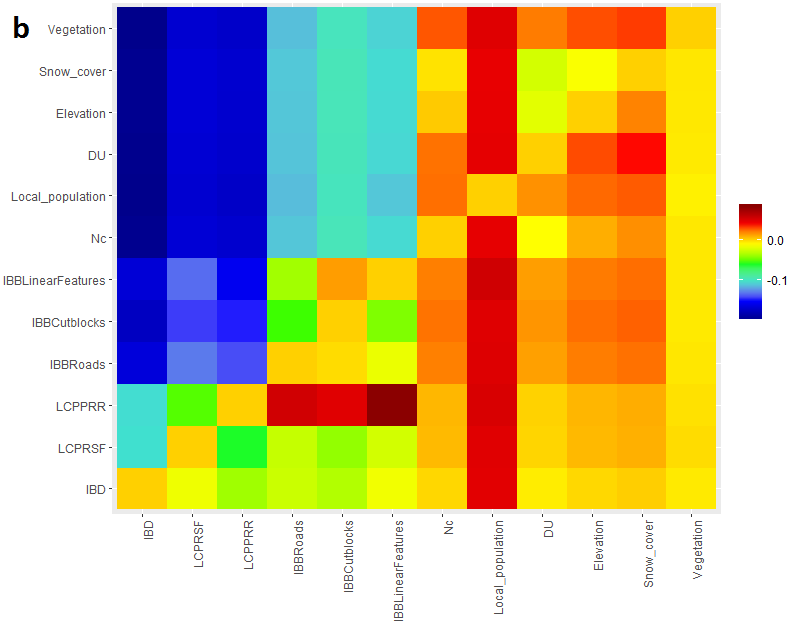


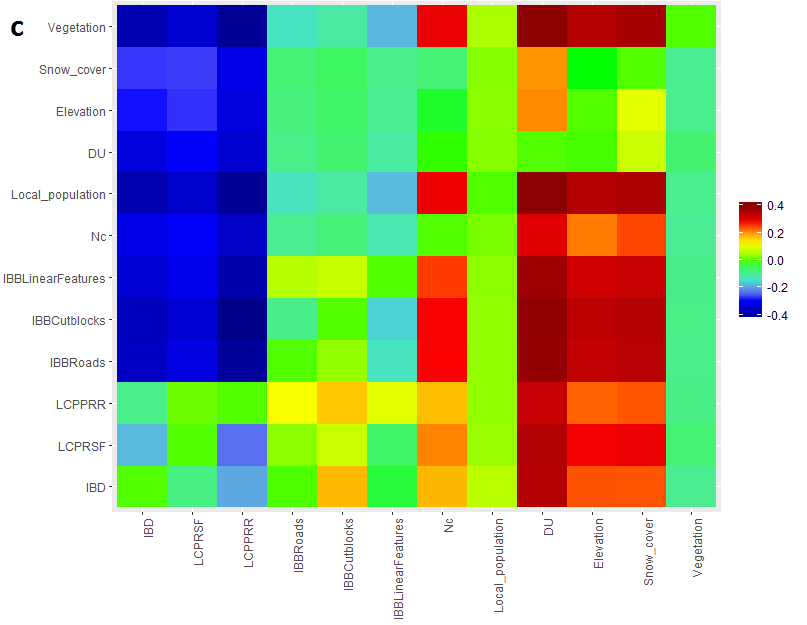

Supplement: Supplementary file 1 [file EVA-10-199-s001.doc]
